# Supplementary material for: Common misconceptions held by health researchers when interpreting linear regression assumptions, a cross-sectional study
Source: PLoS One. 2025 Jun 5;20(6):e0299617. doi: 10.1371/journal.pone.0299617 (PMC12140278; doi:10.1371/journal.pone.0299617)
Supplement: Attachment — Submitted filename: Review PONE-25.docx [file pone.0299617.s004.docx]

**Review PONE-D-24-05674**

**Title: Common Misconceptions Held by Health Researchers When Interpreting Linear Regression Assumptions: A Cross-Sectional Study**

**Authors: Lee Jones, Adrian Barnett, Dimitrios Vagenas**

Dear Editor,

Thank you for the opportunity to review this manuscript. This study offers an insightful exploration of the prevalence and correctness of linear regression assumption checks in health research publications. By identifying common misconceptions and low adherence to statistical best practices, the paper makes an important contribution to the field of meta-research and highlights areas where improvements in statistical literacy and reporting practices are urgently needed.

**Summary of the Manuscript**

The authors analyzed 95 papers published in PLOS ONE in 2019 that utilized linear regression. The results reveal that only 37% of authors checked any linear regression assumptions, and none reported checking all assumptions. A key finding is the widespread misconception that the dependent variable (Y) should be tested for normality instead of the residuals, with only 5 papers correctly addressing residual normality. The study underscores a significant gap in statistical reporting practices and provides actionable recommendations for improving these practices.

**Strengths**

**Relevance**: The paper addresses a critical issue in statistical reporting, which directly impacts the reliability of health research findings.

**Robust Methodology**: The random sampling of papers, dual-statistician reviews, and use of Gwet’s statistic to measure agreement lend credibility to the study.

**Actionable Insights**: The recommendations for teaching, journal reviewing practices, and statistical training are clear and applicable across research domains.

**Suggestions for Improvement**

**Clarity in Statistical Concepts**: The paper could elaborate more on why residual-based checks are critical and provide more detailed explanations of statistical terms like homoscedasticity and independence to ensure accessibility for non-specialist readers.

**Consistency in Terminology**: Terms like "errors" and "residuals" are occasionally used interchangeably. Providing consistent definitions would enhance clarity.

**Detailed Visual Examples**: Including additional visuals, such as examples of incorrect assumption checks and problematic residual patterns, would strengthen the practical impact of the findings.

**Broader Context**: Comparing the findings with reporting practices in other disciplines, such as psychology or social sciences, could help contextualize whether the observed issues are unique to health research or widespread.

**Strengthening Recommendations**: The recommendations could benefit from suggesting specific tools or frameworks (e.g., automated statistical checks, STROBE guidelines) to help authors and reviewers improve adherence to best practices.

**Discussion of Implications**: Expand the discussion on how poor assumption checking impacts the validity of health research outcomes, potentially leading to ineffective or harmful clinical interventions.

While the authors address several limitations, such as the focus on a single journal and variability among statistical raters, they could include suggestions for addressing these issues in future research. Additionally, acknowledging potential improvements in the study design (e.g., inclusion of papers from multiple journals or disciplines) would add transparency.

This manuscript addresses a critical issue in statistical reporting and provides a foundation for improving research practices in health and biomedical sciences. I recommend accepting the paper after minor revisions to enhance clarity, accessibility, and contextual depth.

**Best regards**
