# Supplementary material for: Common misconceptions held by health researchers when interpreting linear regression assumptions, a cross-sectional study
Source: PLoS One. 2025 Jun 5;20(6):e0299617. doi: 10.1371/journal.pone.0299617 (PMC12140278; doi:10.1371/journal.pone.0299617)
Supplement: Attachment — Submitted filename: Response to Reviewers 21 feb.docx [file pone.0299617.s005.docx]

| **Comments from editor** | Thank you for the opportunity to revise our paper. Below, we provide our responses to the nine reviewers' comments.  To enhance accessibility, we have added some of the material previously available on GitHub to the supporting information. We carefully considered the reviewers' feedback and incorporated the constructive suggestions to improve the manuscript. This included restructuring the paper so that the discussion of linear regression is now placed in the introduction. In this section, we have expanded the background on the importance of assumptions, clarified the distinction between residuals and errors, and updated the discussion of statistical assumptions.  Additionally, we have refined the aims and measured outcomes, improved readability by introducing clearer section headings, and rewritten parts of the abstract and conclusion for greater clarity. These revisions enhance the paper’s relevance while ensuring that the material remains accessible to a general audience. |
| --- | --- |
| 1. Please ensure that your manuscript meets PLOS ONE's style requirements, including those for file naming. The PLOS ONE style templates can be found at  [https://journals.plos.org/plosone/s/file?id=wjVg/PLOSOne_formatting_sample_main_body.pdf](https://urldefense.com/v3/__https:/journals.plos.org/plosone/s/file?id=wjVg*PLOSOne_formatting_sample_main_body.pdf__;Lw!!NVzLfOphnbDXSw!GU4WdvIRKIdm7zUEd7uHKkKnsxAdJETE6mJjvyoqfNJJuCxYBGBEp4YUN0gDcc6DwHNMYM7umdT_Gt0TggOu$) and  [https://journals.plos.org/plosone/s/file?id=ba62/PLOSOne_formatting_sample_title_authors_affiliations.pdf](https://urldefense.com/v3/__https:/journals.plos.org/plosone/s/file?id=ba62*PLOSOne_formatting_sample_title_authors_affiliations.pdf__;Lw!!NVzLfOphnbDXSw!GU4WdvIRKIdm7zUEd7uHKkKnsxAdJETE6mJjvyoqfNJJuCxYBGBEp4YUN0gDcc6DwHNMYM7umdT_Gr5VXHZJ$) | We have followed the style guide |
| 2. For ease of reference, please provide a list of the papers assessed in this study. This can be as a file uploaded as supporting information, or as a link included in your revised manuscript text to the location in a repository where this file can be accessed. | Please see the section: Paper selection and randomisation  A complete list of Digital Object Identifiers (DOIs) of included and excluded papers is available for transparency [46].  We have added included papers DOIs and titles as supporting information S2. |
| 2. Thank you for stating the following financial disclosure: | The funding statement has been removed from the manuscript. Please see the updated funding statement.  There was no cost associated with this research except for attending conferences. These costs were covered by the primary author’s PhD allocation from the health faculty, Queensland University of Technology, and scholarships. The Statistical Society of Australia (SSA) and the Association for Interdisciplinary Meta-research & Open Science (AIMOS) supported the primary author with travel grants to attend their respective conferences. The funders had no role in study design, data collection and analysis, decision to publish, or preparation of the manuscript. |
| 4. We are unable to open your figure file [Fig 4.eps and Fig 6.eps]. Please kindly revise as necessary and re-upload. | We have put all the figures through the pace tool and uploaded them. |
| **Review Comments to the Author** |  |
| **Reviewer #1**: The manuscript is based on very unique idea and it is the demand of time to perform study like this. the article is written in very intelligent fashion and all data is available for results reproduction. | Thank you for your feedback |
| **Reviewer #2:** I recommend accepting this paper, but after making the following modifications:  1- I think that the abstract needs improvement. 2- I think that some recent papers related to this research should be mentioned. 3- At the conclusion of this work, the limitations of this research should be mentioned. | Thank you for your feedback. We have rewritten sections of the abstract, updated the limitations, and incorporated more recent references.  While we acknowledge older references in this paper, this reflects the history and key papers in areas such as Altman's 1994: The scandal of poor medical research. There are also many commentaries about poor statistical analysis, but very few undertake primary research to understand the prevalence of reporting linear regression assumptions.  We have added  Pirosca et al. 2022  Knief et.al 2021  Thelwall 2024  Wallisch et.al 2022  Van Aert et al 2023  Castillo et.al 2023  Rahman et.al 2023 |
| **Reviewer #3**: Dear Editor, thank you much for inviting me to review this captivating and very important scientific work. I also appreciate the authors this work. Introduction: This section is looking general and lacks the study’s aims, the linear assumptions. The authors would elaborate the assumptions of linear regression analysis. So the authors should revise and rewrite this section. Research questions: Tense error. Materials and Methods: The first paragraph would be included under introduction section. What it implies in method section? This section does not show the study design in detail, the study variables and measurement. And also it does not data control measures undertaken. Results: The results would be reported according the study’s questions (under three sub-headings) | We have revised the paper to improve its flow and readability. Specifically:   1. Regression description: The regression section has been moved to the introduction, aligning better with the study's context and aims. 2. Study Aims: We have explicitly stated the study aims for clarity. 3. Past Tense for Research Questions: The research questions have been revised to past tense to be consistent with the rest of the paper. 4. Enhanced Headings for Flow: Additional headings have been incorporated to improve the flow of the paper. 5. Subheadings in Results: Two subheadings have been added to the results section, keeping the *reporting of linear regression assumptions* and *misconceptions* together due to their interconnected nature. 6. Agreement of Statistical Raters: This section has been moved to the bottom of the results, now separated with its own heading for clearer distinction. 7. We have included the questionnaire as a supplementary, so it is more easily accessible to readers. This shows what was the statistical reviewers were asked. 8. We have added an outcome measures section. 9. This was a descriptive study with no formal hypothesis tests used with prevalence and 95% confidence intervals reported, while statistical ratings were assessed for reliability.   We hope these changes address your concerns and enhance the clarity and structure of the paper. We appreciate your input. |
| **Reviewer #4**: In my opinion, the paper offers a good review study. I suggest the following modifications: 1. In both equations (Eqs. 1 and 2), the use of symbols with “hat” is incorrect. Why do you use "hat" in the regression equation before estimation? Please check any book on regression models and correct this. 2. The statistical assumptions of the model need to be corrected. 3. One of the important problems in multiple regression is multicollinearity. Why didn't you mention it in this research? Authors should check the multicollinearity test in each paper from the study sample. 4. Add a list of the titles and DOI numbers of papers used in this study to a supplementary file. | Thank you for your feedback. We have added an explanation that statistical inference is used to make conclusions about a population based on sample data. With this context established, we believe the use of hats in our equations is appropriate, as they represent estimated values derived from the sample.  We have also updated the assumptions and added titles and DOI numbers of papers included in the study to the supporting information S2.  We have added a multicollinearity section to the paper (introduction, results and discussion sections) and added two new misconceptions. |
| **Reviewer #5**: General comments: This manuscript contains the results of a reporting review of linear regression usage in PLOS ONE papers published in 2019.  This paper follows in a long line of similar papers that evaluate the usage or reporting of statistical methods. My opinion is that these reviews should be performed regularly and across different research areas. I found this review to be of a high technical standard and feel that it meets the PLOS ONE publication criteria. I have a few general comments below as well as a couple specific comments.  A main point is that information about assumptions being met is predicated on there being the space for and, in my opinion, the desire or requirement of this information to be reported. Regarding the former, in theory, PLOS ONE is a good journal to study because there is no word limit and including supplementary materials is allowed. But, because PLOS ONE is generally not the initial submission choice for authors, some manuscripts may have been initially constructed for journals with word and figure/table limits. I bring this up because, in articles written to a 3,000 or 3,500 word limit, there is rarely the space for a thorough evaluation of the assumptions. In addition, as other methods have become more detailed (e.g., study designs, laboratory methods) the amount of space devoted statistical methods have gotten squeezed out.  This is generally why reviews such as this are performed on more prestigious journals because authors focus on those journals and, hence, the content is more uniform across articles. In addition, more prestigious journals more often have standard statistical review practices. PLOS ONE does have a group of statistical reviewers (of which I am one!), but the sheer volume of papers means we cannot review all of them.  As you mentioned in your limitations, the results are not generalizable to all fields. I will also mention that different fields vary substantially in how and what statistical methods are taught. This is another reason why reviews in field specific journals can be more useful because the typical curriculum for students in that field can be critiqued and recommendations can be made. That can't be done here with the wide array of articles in PLOS ONE.  In addition, reviewers may vary in their statistical knowledge. Although linear models are the cornerstone of statistical modeling, experience may vary substantially. For instance, I have been an applied statistician for over 20 years, but rarely use linear models.  Ultimately, I think reporting is only going to change if journals require it. CONSORT, STROBE, and other reporting guidelines have shown that better reporting can happen. But, authors are not going to change unless failing to report how model assumptions were evaluation will preclude publication. Thus, I think the target audience for your review should be journal editors. Maybe for your next endeavor you can take this article and draft guidelines for the reporting of regression models.  At any rate, I think this is a quality review and I recommend it for publication.  Specific comments: 1. (line 91) The rplos package has been removed from CRAN and archived on GitHub. I think this should be noted in your manuscript since this will make it harder for this research to be reproducible. 2. (line 457) Why is "Questionable Research Practices" capitalized? 3. It wasn't clear to me whether the statisticians read any supplementary materials to evaluate the methods. | Thanks for your interesting comments. It is possible that a lack of space may mean that authors may not report assumption checks that were performed. However, this should not be an issue in *PLOS ONE* with the unrestricted word limit and ability to add multiple supplements. We found that very few authors used these opportunities to report their assumptions, we suspect that this is because most authors do not appreciate the importance of the assumptions. A criterion for publication *in PLOS ONE* is: “Experiments, statistics, and other analyses are performed to a high technical standard and are described in sufficient detail.” So whichever journal the authors previously submitted to, they should have given more information for *PLOS ONE*.  Based on our anecdotal experience, we agree that the training of statistics varies by field. However, we firmly believe that all fields using linear regression must consider the key assumptions.  We agree that reviewers' skills are likely to vary. In future work, we could read the reviewers’ comments to see if there was useful statistical critique.  Response to Specific comments   1. Formerly available versions of the 'rplos' package can still be obtained from the CRAN archive. While the reference cites GitHub, meta-researchers aiming to reproduce this work would already be checking for this detail. Therefore, we do not want to complicate the discussion or detract from our main message that health researchers should be checking regression assumptions. 2. We have removed the capitalisation of questionable research practices 3. We have included: Statistical reviewers were prompted to check the supplementary by asking, "Was most of the detail for the assumption checks in the supporting information (appendix)?"    (1) |
| **Reviewer #6:** The paper offers a good contribution, but I have the following modifications:  1. The "Abstract and Conclusion" sections need improvement; authors should rewrite these sections.  2. There are a few grammatical errors in the paper. The authors need to review the full text carefully.  3. There are several recently published papers that are relevant to this manuscript, so some or all of them should be cited, such as  1. https://doi.org/10.1007/s40520-023-02483-y  2. doi: https://doi.org/10.1080/07853890.2023.2275148  3. doi: https://doi.org/10.1111/jpm.12990  4. doi: 10.1186/s12960-019-0440-y  5. doi: 10.1057/s41599-023-01719-6  6. doi: https://doi.org/10.1063/5.0156458  7. doi: https://doi.org/10.1016/j.ymssp.2022.110022  4. In some equations, some symbols are not defined. Please correct this.  5. Add more statistical details about the linear regression model.  6. Adding future work and the limitations of this research.  7. add the dataset file. | Thank you for your feedback. We have revised the equation's description, added more statistical detail and rewritten the abstract and conclusions. We also updated the limitations and added a future research section. The dataset is available as stated in the data availability section at  <https://github.com/Lee-V-Jones/Reporting_Linear_Regression_Assumptions> |
| Reviewer #8: Summary of the Manuscript  The authors analyzed 95 papers published in PLOS ONE in 2019 that utilized linear regression. The results reveal that only 37% of authors checked any linear regression assumptions, and none reported checking all assumptions. A key finding is the widespread misconception that the dependent variable (Y) should be tested for normality instead of the residuals, with only 5 papers correctly addressing residual normality. The study underscores a significant gap in statistical reporting practices and provides actionable recommendations for improving these practices.  ________________________________________  Strengths  Relevance: The paper addresses a critical issue in statistical reporting, which directly impacts the reliability of health research findings.  Robust Methodology: The random sampling of papers, dual-statistician reviews, and use of Gwet’s statistic to measure agreement lend credibility to the study.  Actionable Insights: The recommendations for teaching, journal reviewing practices, and statistical training are clear and applicable across research domains.  Suggestions for Improvement  Clarity in Statistical Concepts: The paper could elaborate more on why residual-based checks are critical and provide more detailed explanations of statistical terms like homoscedasticity and independence to ensure accessibility for non-specialist readers.  Consistency in Terminology: Terms like "errors" and "residuals" are occasionally used interchangeably. Providing consistent definitions would enhance clarity.  Detailed Visual Examples: Including additional visuals, such as examples of incorrect assumption checks and problematic residual patterns, would strengthen the practical impact of the findings.  Broader Context: Comparing the findings with reporting practices in other disciplines, such as psychology or social sciences, could help contextualize whether the observed issues are unique to health research or widespread.  Strengthening Recommendations: The recommendations could benefit from suggesting specific tools or frameworks (e.g., automated statistical checks, STROBE guidelines) to help authors and reviewers improve adherence to best practices.  Discussion of Implications: Expand the discussion on how poor assumption checking impacts the validity of health research outcomes, potentially leading to ineffective or harmful clinical interventions.  While the authors address several limitations, such as the focus on a single journal and variability among statistical raters, they could include suggestions for addressing these issues in future research. Additionally, acknowledging potential improvements in the study design (e.g., inclusion of papers from multiple journals or disciplines) would add transparency.  This manuscript addresses a critical issue in statistical reporting and provides a foundation for improving research practices in health and biomedical sciences. I recommend accepting the paper after minor revisions to enhance clarity, accessibility, and contextual depth. | We have clarified the difference between errors and residuals and have provided greater detail to give the reader understanding why assumptions need to be checked. In the introduction we outline why poor statistical practice matters and linked it to real world examples. We have included extra citations that show that poor statistical reporting is not unique to the health field.  We have restructured the manuscript, so the explanations of regression, independence and homoscedasticity are in the introduction. We also have updated the limitations section.  Figures 4 (Homoscedasticity) and 5 (linearity) show assumption violations. We have added an example with new figures to the outlier section, with a sensitivity analysis (Fig 6) and an influence plot (Fig 7) to help interpretation.  Have added a paragraph on the SAMPL guidelines to the discussion, which is already recommended at *PLOS ONE.* Automated tools are still under development, with this research rapidly developing with AI, we provide references to up to date discussions on these topics but we are not willing to yet provide a recommendation on tools. This is likely to be future research. |
| **Reviewer #9**: In Linear Regression section:  lines 195 - 197: "... represented as a straight line", this is not always the case, as it oversimplifies the form of linear regression. As has been pointed out, the "linear" term is not for the relationship between the Y and X variables, but between Y and the regression coefficients, which imply that the representation of relationship between X and Y (in the case of 1 predictor) can be of any form, e.g. it can be a bit curvature, for example if the relationship is Y=B0+B1log(X).  Probably it needs more explanation on why the assumption checking is required. In line 201; it is written that linear regression are most commonly fit using OLS. How do you know? Moreover, when using OLS, there is no requirement of the assumptions (unlike if using maximum likelihood estimator), so this might be the case that the majority of authors (of papers examined in this study) were not aware of the assumptions. I suggest mentioning about the Gauss-Markov theorem, that if the OLS estimates fulfill the assumptions, then the BLUE (Best Linear Unbiased Estimators) are obtained, and thus the generalization is allowed.  lines 213 - 214: It is written that the intercept is the estimated value of Y when X=0. While this is mathematically correct, but it is not statistically fully accepted, as this might be misleading, that is interpreting the intercept as the predicted Y-value when X=0, which is a serious pitfall in using regression. If the data does not contain X=0, then the interpretation of the intercept sometimes does not make sense. the intercept is "merely" an adjustment made when estimating the regression parameters so that they will fulfill the condition of SE (sum of error) = 0. | We have expanded our discussion on the importance of assumption checking to clarify why it is a crucial step in regression analysis. However, as our target audience consists primarily of clinicians rather than statisticians, we have tailored the explanation to be accessible and relevant to their needs.  Regarding our statement that linear regression is most commonly fit using OLS, we acknowledge that this is a generalisation. However, OLS remains the default estimation method in most applied clinical research, which aligns with the focus of our study. We have removed the word "most" and included a reference. While we agree that OLS does not require assumptions for estimation per se, the assumptions are necessary for valid inference and generalisation, which is usually the purpose of our target audience. Our paper is aimed at a beginner level; for a more advanced level we have directed people to textbooks.  We appreciate your suggestion to mention the Gauss-Markov theorem, but given our audience, we have chosen to keep the discussion at a conceptual level rather than introducing more advanced theoretical concepts. Instead, we emphasise the practical implications of assumption violations, which are more relevant for clinical researchers interpreting regression results.  Whilst we understand your concern about the intercept, the current interpretation is technically correct and easy to understand. In general, we recommend centering predictors so that the intercept does have a meaning. However, adding this explanation or caveats to the meaning of the intercept is, in our opinion, distracting from getting across the basics that our target audience clearly needs based on the results of our study and prior work. We start this section by stating that it is not an in-depth tutorial on linear regression.  Thank you for your input, which has helped refine our explanations. |
